# Photodynamic Therapy Using IR-783 Liposomes for Advanced Tongue and Breast Cancers in Humans

**DOI:** 10.3390/jfb15120363

**Published:** 2024-12-02

**Authors:** Yasuo Komura, Shintarou Kimura, Yumi Hirasawa, Tomoko Katagiri, Ayana Takaura, Fumika Yoshida, Saki Fukuro, Hiromi Muranishi, Osamu Imataki, Koichiro Homma

**Affiliations:** 1 Rinku Medical Clinic, 2F Medical Rinku Port, 3-41 Rinku Ouraiminami, Osaka 598-0047, Japan; ykomura@rinku-medical-clinic.com (Y.K.); takaura@rinku-medical-clinic.com (A.T.); yoshida@rinku-medical-clinic.com (F.Y.); yanagida@rinku-medical-clinic.com (S.F.); muranishi@kyoto.krg.or.jp (H.M.); 2 StateArt Inc., 2-9-12 Horidome-cho, Nihonbashi, Chuo-ku, Tokyo 103-0012, Japan; s.kimura@stateart.co.jp (S.K.); y.hirasawa@stateart.co.jp (Y.H.); t.katagiri@stateart.co.jp (T.K.); 3 Faculty of Medicine, Kagawa University, 1750-1 Ikenobe, Miki-cho, Kita-gun 761-0793, Japan; imataki.osamu@kagawa-u.ac.jp; 4 Department of Emergency and Critical Care Medicine, Keio University School of Medicine, 35 Shinanomachi, Shinjuku-ku, Tokyo 160-8582, Japan

**Keywords:** breast cancer, heptamethine cyanine dyes, indocyanine green, IR-783, liposome, lung cancer cells, photodynamic therapy, tongue cancer

## Abstract

Photodynamic therapy (PDT) is a minimally invasive treatment that elicits tumor apoptosis using laser light exclusively applied to the tumor site. IR-783, a heptamethine cyanine (HMC) dye, impedes the proliferation of breast cancer cells, even without light. Although studies have investigated the efficacy of IR-783 in cell and animal studies, its efficacy in clinical settings remains unknown. Therefore, we aimed to determine the efficacy of PDT using IR-783 liposomes. An HMC dye, excited by long-wavelength infrared light and with high tissue permeability, was used for PDT after liposomization to enhance tumor tissue accumulation. PDT was performed using IR-783 in two patients with either tongue or breast cancer, one each. IR-783 liposomes inhibited cell proliferation in tongue cancer cells even when not excited by light. Tumor size was markedly reduced in both cases, with no significant adverse events. Furthermore, the patient with tongue cancer exhibited improved respiratory, swallowing, and speech functions, which were attributed not only to the shrinkage of the tumor but also to the improvement in airway narrowing. In conclusion, PDT using IR-783 liposomes effectively reduces tumor size in tongue and breast cancers.

## 1. Introduction

Despite research advances, cancer treatment remains challenging, and its incidence and mortality rates continue to increase worldwide [1]. The complexity of cancer treatment is compounded by several factors, including the presence of metastasis, drug resistance, and treatment discontinuation due to adverse drug events [2]; consequently, photodynamic therapy (PDT) has been developed to address these complex factors. PDT is a highly tumor tissue-specific therapeutic modality that targets cancer cells with internalized photosensitizers [3]. The lack of adverse events associated with the photosensitizers utilized makes PDT an exceptionally noninvasive modality, as it is unlikely to affect normal tissues [3]. Photosensitizers, such as talaporfin sodium or porfimer sodium, excite the surrounding oxygen from its ground state to the singlet state when irradiated with a laser at the sensitizer’s specific excitation wavelength [4]. This oxidant targets lipid membranes and induces ferroptosis in cancer cells as a result of the depletion of reduced glutathione, a substrate of glutathione peroxidase that reduces lipid peroxides in biological membranes to alcohol [5,6,7]. Damage-associated molecular patterns, such as high-mobility group box 1 and adenosine triphosphate, are released from cell membranes disrupted by ferroptosis, which are recognized by the Toll-like receptor family of dendritic cells. The cells subsequently elicit antigen-presenting responses through dendritic cell activation, leading to the activation of cytotoxic T cells [8,9]. Therefore, the induction of ferroptosis by PDT is anticipated to have an antitumor effect not only on light-irradiated tumor tissue but also on cancers that have metastasized throughout the body; this effect will be further enhanced in combination with immune checkpoints [10].

Cytotoxic chemotherapeutic agents, including alkylating agents, platinum drugs, microtubule inhibitors, antimetabolite drugs, and topoisomerase inhibitors, function by impeding cell division and are thus not efficacious against slow-growing cancer cells, such as cancer stem cells [11,12,13]. The efficacy of molecularly targeted drug therapy also depends on the expression of the target enzymes, since the enzymes targeted by existing molecularly targeted drugs are not consistently expressed in cancer cells. Meanwhile, PDT has the potential to be an effective treatment for drug-resistant cancer cells because it can disrupt cancer cells with singlet oxygen [4,5], which in turn induces immunogenic cell death (H-I). In addition, photosensitizers that absorb near-infrared (NIR) light are expected to be effective against cancer cells that are resistant to reactive oxygen species owing to the combined effect of photothermal therapy (PTT) via thermal radiation and the generation of singlet oxygen via photoexcitation [14]. Accordingly, we focused on heptamethine cyanine (HMC) dyes that serve as photosensitizers when excited by NIR light for PDT because longer-wavelength light has the advantage of relatively high tissue penetration [15].

We previously used indocyanine green (ICG) as the HMC for PDT [16] because IR-783 as the HMC has specific cytotoxic activity against breast cancer cells without being influenced by light [17,18]. Low-molecular-weight compounds, such as HMC, facilitate accumulation in solid cancerous tissue when encapsulated in liposomes or micelles [14,19]. This phenomenon, designated as the enhanced permeability and retention (EPR) effect, is characterized by the tendency of macromolecules to leak out of blood vessels and into the tumor stroma owing to the markedly higher vascular permeability of tumor tissue than that of normal tissue [20,21,22].

Despite evidence indicating the efficacy and safety of PDT with IR-783, its efficacy in clinical settings remains unknown. Therefore, this study aimed to determine the efficacy of PDT using IR-783 liposomes in the clinical setting and investigate the cytotoxicity of liposomized HMC in an environment devoid of laser irradiation.

## 2. Materials and Methods

### 2.1. Ethics Approval

This study was approved by the Ethics Review Committee of the IGT Clinic on 24 February 2023 (approval number: 30) and was conducted in accordance with the Declaration of Helsinki and the Ethical Guidelines for Medical Research Involving Human Subjects established by the Japanese Ministry of Health, Labor, and Welfare. Informed consent was obtained from all patients prior to the clinical trial.

### 2.2. Study Design and Patients

This study reported the PDT outcomes in two patients with breast and tongue cancer, one each. Patients with porphyria, with a history of photosensitivity to previous PDT with photosensitizers, such as sodium talaporfin or 5-aminolevulinic acid, were not eligible. The study endpoint was the patients’ ability to receive PDT without photosensitivity reactions or compromising their quality of life.

### 2.3. Preparation of Heptamethine Cyanine Liposomes

ICG and IR-783, as photosensitizers that react with the NIR light used in PDT, were used as HMC dyes. We purchased 1,2-Dimyristoyl-sn-glycero-3-phosphocholine (DMPC) from Yu-ka-Sangyo Co., Ltd. (Tokyo, Japan) and IR-783 from Tokyo Chemical Industry Co., Ltd. (Tokyo, Japan). Diagnogreen (ICG) was purchased from Daiichi Sankyo Co., Ltd. (Tokyo, Japan). Liposomes were prepared as previously reported [16]. Briefly, DMPC was dissolved in a 5% glucose solution at a concentration of 8.85 mM using a Bransonic^®^ CPX8800H-J Ultrasonic Cleaner (Branson Ultrasonic Co., Ltd., Danbury, CT, USA) and sonicated at 40 kHz for 60 min under 45 °C. Subsequently, the liposomes were purified using sterile filtration through a 0.20 μm-pore-size filter (Sartorius, Goettingen, Germany). The particle size of the liposomes was determined through the dynamic light scattering method performed using the ELSZ-2000 (Otsuka Electronics Co., Ltd., Osaka, Japan) apparatus. HMC liposomes were prepared by mixing 5 mg of IR-783 or ICG with 8.85 mM/10 mL of liposomes using a stirrer until the reagent was completely dissolved, followed by sterile filtration through a 0.20 μm filter. HMC liposomalization was determined through gel filtration chromatography using a PD-10 Sephadex G-25 gel filtration column (Cytiva, MA, USA).

### 2.4. Cell Culture

The human lung adenocarcinoma cell line HCC827 was purchased from the American Type Culture Collection (Manassas, VA, USA). Cells were cultured in Roswell Park Memorial Institute 1640 (RPMI 1640) medium (Merck & Co., Inc., Rahway, NJ, USA) supplemented with 2 mM L-glucose, 10% fetal bovine serum, and 100 U/mL penicillin-streptomycin at 37 °C in 5% CO_2_.

### 2.5. Proliferation Measurement

HCC827 cells were seeded in 12-well plates at a density of 1 × 10^4^ cells per well. After 24 h, cells were treated with ICG, ICG liposomes, IR-783, and IR-783 liposomes with 10 μM of the respective HMC equivalents, and cell growth was monitored using an incubation monitoring system (Olympus CM30).

### 2.6. MTT Assay

Cell viability was determined by adding 5 mg/mL of tetrazolium salt, 3-(4,5-dimethylthiazol-2-yl)-2,5-diphenyltetrazolium bromide (MTT), to the culture medium of HCC827 cells followed by incubation at 37 °C for 3 h. Subsequently, the medium was removed, and the cells were lysed with 500 μL of dimethyl sulfoxide. Absorbance was measured at 570 nm.

### 2.7. IR-783 Liposome Therapeutic Intervention

IR-783 liposomes were diluted to 50 mL with 5% glucose (Hikari Seiyaku Co., Ltd., Tokyo, Japan) and infused intravenously at a rate of 2 mL/min through a vein in the middle of the patient’s arm. A multilaser delivery system (Weber, Germany) was selected as the laser delivery device for PDT. PDT was performed at a laser power density of 25 mW/cm^2^ using a fiber with a wavelength of 810 nm approximately 24 h after liposome administration.

### 2.8. PDT for Tongue Cancer

The outer circumference of the MLDS infrared fibers was wrapped with a wrapping film, and the patient was instructed to lightly bite the portion of the fiber that was in contact with the mantle of the syringe. Irradiation with an 810 nm laser for 20 min was subsequently performed. A nasal cannula (MC Medical, Inc., Tokyo, Japan) was attached to a valve with a medical oxygen flow regulator (Air Liquide Japan G.K., Tokyo, Japan), and oxygen was administered intranasally to the patient at approximately 2 L/min during the intervention.

### 2.9. PDT for Breast Cancer

Five MLDS infrared fibers were passed through the aperture in the masher, wrapped in a wrapping film. Irradiation with an 810 nm laser for 40 min at the limit of contact with the lesion was then performed.

### 2.10. Statistical Analysis

All results are expressed as the mean ± standard deviation. The statistically significant differences were analyzed using the one-way analysis of variance followed by Bonferroni’s post hoc test. All statistical analyses were performed using the add-in software Statcel4 (v4.0; OMS Publishing, Inc., Tokorozawa, Japan). Significance levels were set at *p* < 0.05 and *p* < 0.01.

## 3. Results

### 3.1. T Physicochemical Characteristics of HMC Liposomes

**Figure 1:**
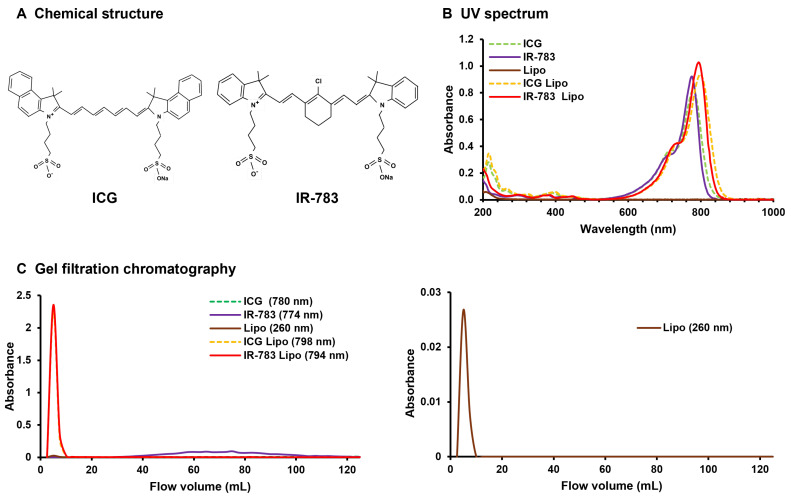
(**A**) Chemical structure of ICG and IR-783. (**B**) UV spectrum results. The light-grey dotted line depicts ICG in isolation, whereas the straight line represents IR-783. The dark grey straight line represents liposomes alone, the black dotted line represents ICG liposomes, and the straight line represents IR-783 liposomes. The absorbance peaks of both ICG and IR-783 tend to shift to the right when liposomes are used. (**C**) Gel filtration chromatography results. The light-grey dotted line depicts ICG in isolation, whereas the straight line represents IR-783. The dark-grey line represents liposomes alone, the black dotted line represents ICG liposomes, and the straight line represents IR-783 liposomes. The left side shows all liposome types, whereas the right side shows single liposomes with low absorbance values. ICG could not be quantified at the limit of detection, and IR-783 was only sporadically detected in the latter half of the sample owing to its small size. Both liposomized ICG and IR-783 are observed at the same locations as the standalone liposomes rather than at the sites where they are detected independently. This finding confirms that ICG and IR-783 are liposomized. ICG: indocyanine green; Lipo: liposomes.

The chemical structures of ICG and IR-783 are illustrated in Figure 1A. IR-783 showed higher NIR absorption than ICG (Figure 1A), consistent with previous reports [23]. Moreover, both liposomalized IR-783 and ICG demonstrated enhanced absorption in the NIR region after processing (Figure 1B). The absorption wavelength of ICG liposomes undergoes a slight shift towards longer wavelengths because of the dissolution of ICG within the hydrophobic groups of the phospholipid bilayer [24]. Here, this phenomenon occurred not only with ICG but also with IR-783 (Figure 1B). Encapsulation of HMC in liposomes was investigated using gel filtration chromatography. The wavelengths of ICG, ICG liposomes, IR-783, and IR-783 liposomes, as determined by gel filtration chromatography, were identified based on the UV spectrum results. However, only the liposomes are depicted on the right side of Figure 1C for clarity. Smaller particles were identified in subsequent fractions in gel filtration chromatography. ICG was below the limit of detection, whereas IR-783 was detected in an inconsistent manner across the 40–100 mL fractions (Figure 1C). The same fractions in which liposomes were detected prior to inclusion contained both liposomized ICG and IR-783 (Figure 1C). A size of 5–100 nm is considered optimal for the EPR effect to accumulate macromolecules in tumor tissues [22] because this range is not subject to renal clearance. Additionally, a particle size as small as 30 nm was recently identified as particularly effective, as it can easily pass through the cancer stroma [25]. Accordingly, we modified liposomes to a diameter of approximately 20 nm (Table 1). Both ICG and IR-783 exhibited a change in charge upon liposomization, with a positive tilt (Table 1). Moreover, the incorporation of HMCs into liposomes has been hypothesized to modify the surface potential. The results of our UV spectroscopy, gel filtration, and zeta potential analyses demonstrated that the IR-783 liposomes could effectively encapsulate IR-783.

### 3.2. Inhibitory Effect of IR-783 Liposome Treatment on Cell Proliferation

**Figure 2:**
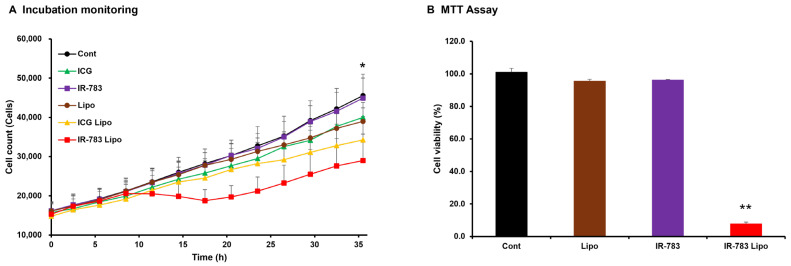
(**A**) Result of real-time measurement of HCC827 cell proliferation over 36 h using an incubation monitoring system. HMC liposomes were treated with cells at equivalent concentrations to the respective HMC (10 μM), while controls and liposomes were treated at the same volume as the HMC (100 μL). The white circles represent the control group (5% glucose), while the black circles indicate the liposome group. The white triangles represent the ICG group, and the black triangles indicate the liposome group. The white squares represent the IR-783 group, and the black squares indicate the IR-783 liposome group. The vertical axis represents the number of cells, while the horizontal axis depicts time. Significant discrepancies in cellular proliferation after 36 h were determined exclusively between the control and IR-783 liposome groups. (**B**) Results of MTT assay for measuring the viability of HCC827 cells treated with IR-783 and IR-783 liposomes at 10 μM of IR-783 equivalent after 48 h. The controls and liposomes were treated with 100 μL, which was the same volume as that used for IR-783 and IR-783 liposomes. The cells treated with IR-783 liposomes show significantly lower viability compared with the control cells. The results were analyzed via one-way analysis of variance with Bonferroni’s post-test and are expressed as the mean ± standard deviation (*n* = 5, * *p* < 0.05, ** *p* < 0.01). ICG: indocyanine green; Lipo: liposomes.

The inhibitory effect of HMC and HMC liposomes on the proliferation of HCC827 lung-cancer-derived cells was observed in real time using a culture monitoring system (Figure 2A). The results demonstrated that only IR-783 liposomes significantly inhibited HCC827 cell proliferation at 36 h post culture. Similarly, ICG liposome-treated cells better inhibited cell proliferation compared to the control, although the difference was not significant. Furthermore, the inhibitory effect of IR-783 liposomes on cell growth was evaluated using the MTT assay (Figure 2B). The results were comparable to those in the monitoring system, except in cells treated with liposomal IR-783 that exhibited significantly lower viability than the control group did.

### 3.3. PDT Using IR-783 Liposome for the Patient with Tongue Cancer

**Figure 3:**
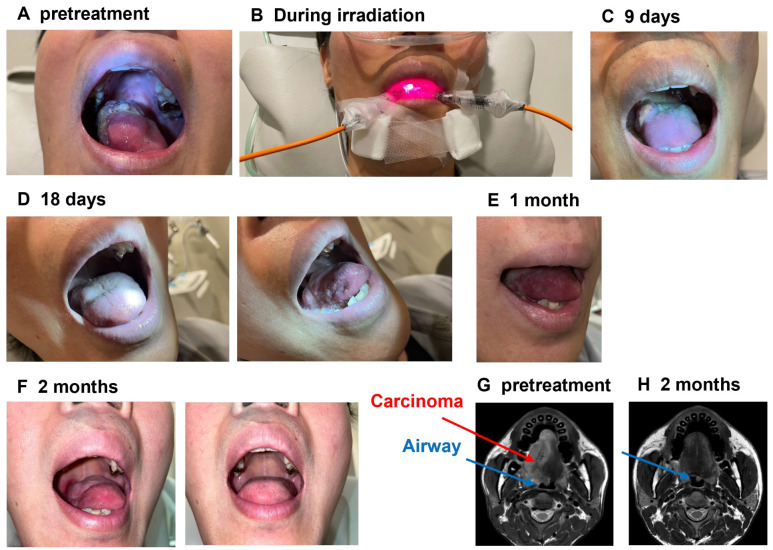
(**A**) Images showing the patient with tongue cancer prior to photodynamic therapy (PDT) using IR-783. (**B**) The patient undergoing infrared light irradiation. A large tumor is visible on the right middle part of the tongue. (**C**) Image obtained 9 days following the initial intervention, showing a reduction in tumor size. (**D**) Image obtained 18 days following the initial intervention, showing that the tumor in the upper portion of the tongue was no longer discernible, and only an ulcerative lesion in the right side of the tongue was visible. (**E**,**F**) Images obtained 1 and 2 months following the initial intervention, respectively. No lesions were identified, and no observable tendency for recurrence was noted. (**G**) Magnetic resonance imaging (MRI) of the patient prior to PDT using IR-783. (**H**) MRI of the patient 2 months after the initial intervention. The red line denotes the tumor tissue, while the blue line represents the airway. After 2 months, the tumor was no longer discernible on the MRI, and the stenotic airway was recovered.

Despite being diagnosed with tongue cancer in 2022, the male patient in his 30s did not pursue professional cancer treatment and thus had disease progression without intervention. In December 2023, the lesion advanced, extending to the upper palate and causing respiratory distress (Figure 3A,G). Consequently, the patient sought phototherapy in our hospital. In February 2024, 5 mg of IR-783 liposomes was administered intravenously at an IR-783 equivalent dose, and PDT was performed the following day at 60 J/cm^2^ using two 810 nm probes (Figure 3B). We decided to increase the IR-783 dosage owing to the improvement in lesions, reduction in pain, and normal biochemical test results after PDT. At 1 week after the initial dose, IR-783 liposomes (10 mg in 783 equivalents) were intravenously administered over 2 days. On days 2 and 3, we performed PDT under conditions identical to those used for the initial dose. After three phototherapy sessions, the tumor in contact with his upper palate started to shed partially (Figure 3C), and he reported an improvement in his breathing. Eighteen days after the initial intervention, the tumor in the upper portion of the tongue regressed, leaving only an ulcerative lesion on the right lateral aspect of the tongue (Figure 3D). After 1 month, the ulcerated area had disappeared (Figure 3F), and magnetic resonance imaging (MRI) T2 scans taken 3 months later showed no lesions. Additionally, an improvement in airway narrowing was observed after 2 months compared with the initial scan (Figure 3G,H).

### 3.4. PDT Using IR-783 Liposome for the Patient with Breast Cancer

**Figure 4:**
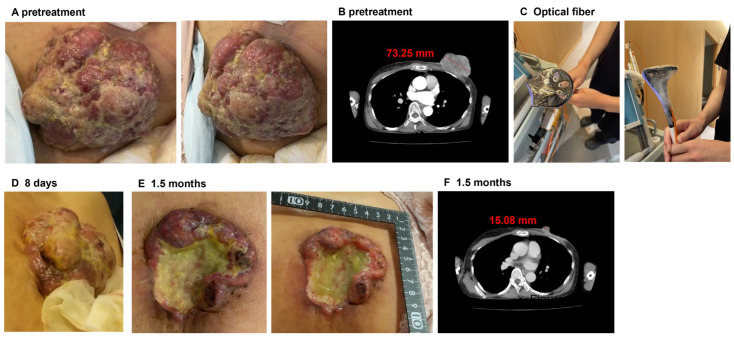
(**A**) Pretreatment image of the tumor in the breast cancer patient. A large-sized tumor was observed in the left breast. (**B**) Pretreatment CT image. (**C**) Image of the irradiation equipment used in PDT. Five optical fibers for irradiation were grouped together in a washer and wrapped with wrapping film. (**D**) Image captured 7 days after the first treatment. The tumor was slightly smaller compared to before PDT. (**E**) Image obtained approximately 1.5 months after the initial therapeutic intervention. The tumor size was markedly reduced. (**F**) CT image obtained 1.5 months after the initial treatment. The tumor was markedly reduced.

A woman in her 60s was diagnosed with breast cancer in 2015 and was initially treated with a combination of taxane and pembrolizumab. In 2022, the patient developed massive breast discharge and pelvic metastasis and required radiation therapy for pain relief. However, in 2023, her physician determined that the pharmacological intervention was ineffective and thus withdrew treatment. In May 2024, she was admitted to our hospital because of the lack of alternative treatment options for her medical condition following the cessation of medication. At the time of presentation, computed tomography (CT) confirmed the presence of a mass in her left breast (Figure 4A,B). With her informed consent, we decided to perform PDT with IR-783 liposomes because the lesion was visible. On the first 2 days, 12.5 mg of IR-783 liposomes were intravenously administered at an equivalent dose of IR-783, and PDT at 300 mJ/cm^2^ was subsequently performed using five 810 nm fibers wrapped in wrapping film in the following 2 days (Figure 4C). During PDT, the lesion exhibited significant exudate secretion; however, the patient did not report any pain. Exudation immediately ceased after the initial PDT, and over the course of 1 week, the lesion surface slowly turned blackish and dry. As the lesion improved (Figure 4D), we administered IR-783 liposomes on day 8 for 2 more days under the same conditions, followed by two additional PDT sessions. When she returned to our clinic approximately 6 weeks later, CT imaging showed that the raised mass previously observed had been dislodged and had significantly decreased in size compared with the initial presentation (Figure 4E-F).

## 4. Discussion

This study aimed to determine the efficacy of PDT using IR-783 liposomes in the clinical setting and investigate the cytotoxicity of liposomized HMC in an environment devoid of laser irradiation. Our findings indicate that using IR-783 liposomes as photosensitizers for PDT can impede the proliferation of cancer cells without the need for photoexcitation with antitumor effects against both tongue and breast cancers. Although several anti-tumor effects of PDT and PTT using IR-783 have been documented in cell and animal studies [26,27,28], to the best of our knowledge, this is the first clinical report of PDT using IR-783 in humans.

IR-783 can inhibit the growth of cancer cells in the absence of laser irradiation [17,18]. However, our results demonstrated that liposomalized IR-783, but not IR-783 alone, had a notable inhibitory effect on the proliferation of lung cancer cells (Figure 2). This discrepancy may be because previous studies required treatment of cells with IR-783 at concentrations of 100 μM or higher to achieve cell death [17,18], in contrast to our study, which used a significantly lower concentration of 10 μM. The previous findings indicate that although the survival rate in breast cancer cells treated with IR-783 at concentrations below 10 μM was reduced, it was largely unaltered when compared to that in control cells [17,18]. The inhibitory effects of both IR-783 and ICG on cancer cell growth through liposomalization (Figure 2) are because of the enhanced efficacy of these HMCs owing to increased cellular uptake facilitated by liposomalization. In this clinical trial, PDT was performed with IR-783 liposomes as these liposomes demonstrated a superior anti-tumor effect compared to ICG liposomes.

The patient with tongue cancer presented with various symptoms, including dyspnea, dysphagia, dysgeusia, taste disorders, and speech disorders, all of which were attributed to the tumor. Pre-treatment MRI observations indicated that surgical intervention and radiotherapy were unlikely to be effective in improving cancer-related complications. However, lesion size was markedly reduced with PDT treatment (Figure 3), which in turn improved his breathing, taste, and speech functions. These findings indicate that PDT may represent a promising modality for the future treatment of tongue cancer. Additionally, the patient with breast cancer who previously demonstrated no improvement with drug therapy exhibited a notable reduction in tumor size following the PDT intervention (Figure 4). These findings suggest that PDT using IR-783 liposomes may be an effective treatment for tumors resistant to cytotoxic chemotherapeutic agents and immune checkpoint inhibitors. Although not reported in this report, the patient’s lesions are improving and will continue to be monitored and, if necessary, treated in the same manner.

Despite the evidence indicating the clinical efficacy and safety of PDT with IR-783, several challenges remain unresolved. The precise mechanism by which IR-783 exerts its antiproliferative effects remains to be elucidated and requires further investigation to gain a deeper understanding of this process. Future studies should investigate the previously reported mitochondrial mitogenic effect of IR-783 building on the enhancement of endocytosis through IR-783 liposomalization in this study [17,18]. Additionally, we aim to expand the scope of our research in the future through collaboration and other means as more patients are accumulated to overcome the small sample size limitation in this study. We also intend to conduct further statistical analyses, including 5-year survival rates. Moreover, invasive procedures, such as laparotomy and thoracotomy, are required to treat visceral tumor tissues with PDT, given that this treatment is essentially aimed at tumor tissue confined to the superficial layers of the body. Such a procedure would be challenging to perform in a clinic of our size and would require the resources of a larger hospital. Furthermore, sonodynamic therapy (SDT), which employs ultrasound waves that penetrate deep into the body to stimulate photosensitizers, has garnered considerable attention in recent years [29]. The inhibitory effect of SDT with IR-780, a derivative of IR-783, on cancer cells was demonstrated in both cell and animal experiments [29,30]. Consequently, further studies are planned to investigate the potential of SDT using IR-783 as a treatment modality for pancreatic and liver cancers at our clinic.

## 5. Conclusions

This study demonstrates that IR-783 liposomes inhibit cell growth and shrink tongue and breast cancer cells. The utilization of IR-783 as a photosensitizer for PDT is anticipated to represent a novel approach to cancer eradication, particularly for patients with refractory advanced cancer, without imposing an undue burden.

## Figures and Tables

**Table 1:** Physicochemical characteristics of HMC liposomes.

Sample	Diameter (nm)	Zeta Potential (mV)
ICG	N.D.	−33.9 ± 3.46
IR-783	N.D.	−18.8 ± 3.57
Lipo	25.8 ± 1.42	8.4 ± 0.77
ICG Lipo	20.1 ± 0.83	−9.0 ± 5.38
IR-783 Lipo	21.8 ± 1.65	−9.5 ± 0.41

The results are expressed as the mean ± standard deviation (*n* = 3). ICG: indocyanine green; Lipo: liposomes.

## Data Availability

The original contributions presented in the study are included in the article, further inquiries can be directed to the corresponding author (K.H.).
